# Advice About Screening for Prostate Cancer With Prostate-Specific Antigen

**Published:** 2017-09-01

**Authors:** Gannel Jean-Pierre

**Affiliations:** Stamford, Connecticut

Prostate cancer is the second-leading cause of cancer death in male Americans, behind only lung cancer. It is estimated there will be 161,360 new cases and 26,730 deaths due to prostate cancer in 2017 (American Cancer Society [ACS], 2017). There are several treatment options for patients diagnosed with prostate cancer, such as active surveillance, surgery, hormone therapy, radiation therapy, chemotherapy, and immunotherapy. In addition, adequate follow-up, further diagnostic tests, and effective management of prostate cancer are available and accessible.

Advising a patient about screening for prostate cancer with prostate-specific antigen (PSA) represents a challenge. Clinicians vary in their opinions on advising their patients about screening for prostate cancer; some believe that finding and treating prostate cancer early may save lives, whereas others maintain that prostate cancer may never affect a man’s health (Basch et al., 2012; Centers for Disease Control and Prevention [CDC], 2013).

## PROSTATE-SPECIFIC ANTIGEN

The PSA test was approved in 1986 by the US Food and Drug Administration (FDA) to monitor the progression of the disease in men diagnosed with prostate cancer. In 1994, it was approved by the FDA to be used along with a digital rectal exam (DRE) to help detect prostate cancer in asymptomatic men (National Cancer Institute [NCI], 2017). 

Prostate-specific antigen is called a biologic or tumor marker, which is defined by the NCI (n.d.) as a biologic molecule found in blood, other body fluids that indicates a normal or abnormal process, or of a condition or disease. Normal and cancerous prostatic epithelial cells secrete the glycoprotein PSA. Thus, PSA is highly specific for the prostate. It is usually found in elevated levels in the serum of men with prostate cancer. However, a low level of PSA does not guarantee an absence of cancer (ACS, 2016).

## SCREENING FOR PROSTATE CANCER

The Occupational Safety and Health Administration (n.d.) defines the fundamental purpose of medical screening as early diagnosis and treatment. Wald (2008) defines medical screening as a process of selection with the purpose of identifying people at high risk to develop a specific disorder. In a stricter sense, Wilken et al. (2012) define medical screening as a method for detecting disease or body dysfunction before a person would normally seek medical care. 

Effective medical screening follows certain criteria; for example, the condition sought should be an important health problem, and there should be an accepted treatment for those with the recognized disease (Holland, Stewart, & Masseria, 2006; Wilken et al., 2012). One can argue that screening for prostate cancer meets the two suggested criteria since prostate cancer is an important health problem and several treatment options exist. 

A screening test must also meet certain criteria. Holland, Stewart, and Masseria (2006) suggest that a screening test must be simple to perform, easy to interpret, acceptable to those undergoing it, accurate, and repeatable. Based on these criteria, a desirable screening test for prostate cancer should be sensitive, specific, cost-effective, valid, reliable, easy to administer, and should allow treatment to start at a stage when intervention reduces morbidity and mortality. The PSA test is easy to administer and cost-effective. It is also repeatable. However, its accuracy is controversial. 

## PSA CONTROVERSY

Although PSA is highly specific for the prostate, it is not prostate cancer–specific, since PSA serum in the blood may be increased in many situations, such as prostate cancer, urinary retention, benign prostatic hyperplasia, DRE, ejaculation, perineal trauma, prostate biopsy, prostate surgery, and prostatitis, and may be decreased by approximately 50% in patients taking 5-alpha reductase inhibitors such as finasteride and dutasteride (National Comprehensive Cancer Network [NCCN], 2016). Moreover, screening with PSA can often lead to overdiagnosis and consequently overtreatment by detecting cancerous tumors that are not life threatening, do not need treatment, and can be managed by active surveillance (NCI, 2017). There is convincing evidence that PSA-based screening programs result in the detection of many cases of asymptomatic prostate cancer and that a substantial percentage of men who have asymptomatic cancer detected by PSA screening have a tumor that either will not progress or will progress so slowly it would have remained asymptomatic for the man’s lifetime (US Preventive Services Task Force [USPSTF], 2013). 

Eckersberger et al. (2009) suggest that compared with the large number of men diagnosed and treated for prostate cancer, the declines in mortality are quite small. They also report that the diagnosis of prostate cancer causes anxiety, which is the main reason for seeking active treatment in patients with clear indolent cancer.

Results from the European Randomized Study of Screening for Prostate Cancer (ERSPC; 2009) conducted in 7 European centers found that 1,410 men would need to be screened approximately twice over a period of 9 years to prevent a single death. The results of the prostate cancer portion of another trial called the Prostate, Lung, Colorectal, and Ovarian (PLCO) Cancer Screening Trial show that men in the screening group had a higher incidence of prostate cancer and that mortality was not reduced in the screening group compared with the control group (Andriole et al., 2012). The NCI (2016) suggests that the evidence is insufficient to determine whether screening for prostate cancer with PSA results in a reduction of mortality from prostate cancer. 

## PSA TEST RESULT INTERPRETATION

The NCI (2017) reports no specific normal or abnormal level for PSA. But most clinicians in the past considered PSA levels of 4.0 ng/mL and lower as normal. With a PSA between 4.0 and 10.0 ng/mL, men have about a 1 in 4 chance of having prostate cancer and over a 50% chance if the PSA level is more than 10.0 ng/mL (ACS, 2016). However, it is reported that 15% of men with a PSA number below 4.0 ng/mL actually have prostate cancer. Consequently, several clinicians are now using a threshold range of 2.5 to 3.0 ng/mL for proposing prostate biopsy; men with a PSA of less than 2.5 ng/mL may only need to be retested every 2 years and yearly if they have a PSA of 2.5 ng/mL or higher (ACS, 2016). 

The NCCN (2016) guidelines recommend repeat testing at 2- to 4-year intervals for a PSA < 1.0 ng/mL with a normal DRE (if done); repeat testing at 1- to 2-year intervals for a PSA of 1.0 to 3.0 ng/mL with a normal DRE (if done); and a referral for biopsy for a PSA > 3.0 ng/mL for men between 45 and 75 years old. Men aged ≥ 60 years with a serum PSA < 1.0 ng/mL have a very low risk of metastases and may not benefit from further testing. A PSA cutoff point of 3.0 ng/mL at age 75 years also has a low risk of poor outcome. If PSA level is < 3.0 ng/mL with a normal DRE (if done) for men older than age 75, and if there is no other indication for biopsy, repeat testing should be done at 1- to 4-year intervals. 

## CONSEQUENCES OF AN ELEVATED PSA

When an asymptomatic patient has an elevated PSA serum, his clinician may advise him to repeat the test after waiting for a length of time or to undergo a prostate biopsy to rule out prostate cancer. The NCI (2017) indicates that no clear consensus has been found on the optimal PSA level for recommending a prostate biopsy for men of any racial or ethnic background. 

The NCCN (2016) recommends referral for biopsy for a PSA level > 3 ng/mL for men between 45 and 75 years old. However, the majority of panel members agree that a decision to perform a biopsy should not be based on a PSA cutoff point alone but should incorporate other important clinical variables, including age, family history, PSA kinetics, race, health status, and patient preference. Undergoing biopsy can be stressful, and some men have persistent anxiety regarding possible cancer despite negative biopsy results. 

The USPSTF (2013) reports up to one-third of men undergoing biopsy will experience fever, infection, bleeding, urinary problems, pain that they consider a moderate or major problem, and that 1% will be hospitalized for these complications. Some clinicians, in accordance with their patients, might consider using biomarker testing to further define the probability of cancer before proceeding to biopsy and its associated risks. 

## BIOMARKERS BEING STUDIED TO IMPROVE PSA TESTING

The NCI (2017) presents some biomarkers being studied to improve PSA testing. In the measurement of free PSA (fPSA) vs. total PSA (tPSA), where fPSA is the amount of PSA that is not bound to other proteins divided by tPSA, a lower proportion of fPSA may be indicative of a more aggressive cancer. The PSA density of the transition zone of the prostate (the interior part of the prostate that surrounds the urethra) is the blood level of PSA divided by the volume of the transition zone of the prostate. This measure may be more accurate than the standard PSA test at detecting prostate cancer. Age-specific PSA reference ranges that take into consideration that PSA level tends to increase with age may increase the accuracy of the PSA test. The PSA velocity (the change in PSA level over time) and the PSA doubling time (the time it takes for the PSA level to double) may be helpful in predicting prostate cancer. ProPSA, which refers to several inactive precursors of PSA, is more strongly associated with prostate cancer than with benign prostatic hyperplasia. 

The NCCN (2016) panel recommends the following biomarkers capable of improving the specificity of the PSA test. The percent free PSA (%f PSA; the percentage of the unbound form of PSA in the blood) is a clinically useful molecular form of PSA with the potential to improve early detection staging and monitoring of prostate cancer. It is significantly lower in men who have prostate cancer compared with men who do not.

The Prostate Health Index (PHI; a combination of tPSA, fPSA, and proPSA) was noted to have approximately double the sensitivity of fPSA/tPSA for cancer detection in those with serum PSA concentrations between 2.0 and 10.0 ng/mL.

The 4Kscore test is another combination test that measures fPSA, tPSA, human kallikrein 2 (hK2), and intact PSA and also considers age, DRE results, and prior biopsy status. This test reports the percent likelihood of finding the high-grade (Gleason score ≥ 7) cancer on biopsy.

The ConfirmMDx test is a tissue-based, multiplex epigenetic assay with the goal to improve the stratification of men being considered for repeat prostate biopsy, since it may identify individuals at higher risk of prostate cancer diagnosis on repeat biopsy. Prostate cancer antigen 3 (PCA3) is a noncoding prostate tissue–specific RNA that is overexpressed in prostate cancer. Current assays quantify PCA3 overexpression in post-DRE urine specimens. It appears to be more useful in determining which patients should undergo a repeat biopsy. The FDA has approved the PCA3 assay to help decide, along with other factors, whether a repeat biopsy in men 50 years or older with one or more negative prostate biopsies is necessary. Thus, the %f PSA, the PHI, and the 4Kscore may be considered for patients with serum PSA levels > 3.0 ng/mL and for those who have not yet had a biopsy. The %f PSA, the PHI, the 4Kscore, the PCA3, and the ConfirmMDx test may also be considered for men who have had at least one prior negative biopsy and are thought to be at higher risk.

## DIAGNOSING PROSTATE CANCER BY BIOPSY 

Men diagnosed with prostate cancer by biopsy should be managed according to the NCCN (2016) treatment guidelines for prostate cancer. Among men diagnosed with cancer on prostate biopsy, the panel does not recommend routine repeat biopsy except in special circumstances, such as on the suspicion the patient harbors more aggressive cancer that was evident on the initial biopsy and the patient is otherwise a candidate for active surveillance as outlined in the treatment guidelines. The NCCN (2016) suggests that a negative prostate biopsy does not preclude a diagnosis of prostate cancer on subsequent biopsy. Consequently, those patients should be followed with repeat DRE and PSA at 6- to 24-month intervals, with repeat biopsy based on risk. Biomarker testing for biomarkers such as fPSA, PCA3, 4Kscore, PHI, and ConfirmMDx may be considered. 

In addition, the NCCN (2016) reports that approximately 10% of patients undergoing biopsy will be found to have high-grade prostatic intraepithelial neoplasia (HGPIN). Cytologically, the nuclear features of HGPIN resemble those of malignant tumors; however, the presence of a basal layer on the acini distinguishes this entity from cancer.

Focal HGPIN should be managed in the same manner as a benign result. If it is multifocal (> 2 sites), an extended pattern rebiopsy is recommended within 6 months, with increased sampling of the affected site and adjacent areas. If no cancer is found, close follow-up with PSA and DRE is recommended at 1-year intervals initially. Atypia that is suspicious for cancer and characterized by small single-cell layer acini represents one of two possibilities: normal prostate tissue distorted by an artifact or prostate cancer that does not meet the histologic criteria for a diagnosis of prostate cancer. Men with atypia suspicious for cancer should be managed in the same manner as men with multifocal HGPIN (NCCN, 2016).

## EXPERT RECOMMENDATIONS ABOUT SCREENING FOR PROSTATE CANCER

The USPSTF (2013) recommends against PSA-based screening for prostate cancer. This recommendation applies to men in the general US population regardless of age and is based on various studies such as the US PLCO and the ERSPC trials and a study by Chou et al. (2011), which concluded there is a small or no reduction in prostate cancer–specific mortality related to PSA-based screening and it is associated with harms related to subsequent evaluation and treatment, some of which may be unnecessary. There is convincing evidence that PSA-based screening results in the detection of many cases of asymptomatic prostate cancer that will not progress or will progress so slowly the individual would probably die of other causes.

The American Academy of Family Physicians (AAFP) recommends against PSA-based screening for prostate cancer because the harms outweigh the benefits in most men (Mulhem, Fulbright, & Duncan, 2015). Most men who are found to be positive for prostate cancer through screening do not necessarily benefit from screening since their tumors are not aggressive. Clinicians should inform patients about the risks and benefits of PSA-based screening using shared decision-making. Prostate cancer screening should not be performed in men younger than 50 years or older than 70 years or in men with a life expectancy of less than 10 to 15 years (Mulhem et al., 2015). 

The NCI (2016) suggests there is insufficient evidence to determine whether screening for prostate cancer with PSA results in a reduction of mortality from prostate cancer. The NCI also suggests that PSA-based screening for prostate cancer has led to some degree of overtreatment and adverse psychological effects in men who have a prostate biopsy without identified prostate cancer. The NCI (2017) suggests that clinicians should instruct any man considered for prostate cancer screening about its potential harms and benefits. 

The CDC (2016) follows the USPSTF recommendations against PSA-based screening for men who do not have symptoms. However, the CDC continues supporting informed decision-making, understanding that men and their clinicians may decide to continue to screen for prostate cancer. 

The ACS (2016) guideline for prostate cancer screening recommends shared decision-making as a prerequisite to screening. Men should be instructed about the uncertainties, risks, and potential benefits of prostate cancer screening before making an informed decision with their clinicians about whether to be screened for prostate cancer. The discussion about prostate cancer screening should take place at age 50 for men who are at average risk with a life expectancy of at least 10 more years; at age 45 for men at high risk such as black men and/or men with a first-degree relative who was diagnosed with prostate cancer before 65 years of age; and at age 40 for men at an even higher risk (those with more than one first-degree relative who had prostate cancer at an early age). Finally, asymptomatic men with a life expectancy of less than 10 years should not be offered testing, since they are not likely to benefit from it.

The American Urological Association (AUA) recommends against PSA screening for prostate cancer in men younger than age 40 (2013). Routine screening in men between the ages of 40 and 54 years at average risk is not recommended. Shared decision-making for men between the ages of 55 and 69 years who are considering PSA screening is recommended. The panel does not recommend routine PSA screening in men older than age 70 or in any man with less than a 10- to 15-year life expectancy (AUA, 2013). The new guidance moves the AUA significantly closer to the position against all PSA screening set out this past year by the AAFP and the USPSTF.

The NCCN (2016) recognizes prostate cancer could be a life-threatening disease and that not all men diagnosed with prostate cancer should be treated. The guidelines recommend clinicians to start a discussion with their patients about the risks and benefits of offering baseline PSA levels between the ages of 45 and 75 years. Healthy men older than age 75 with little or no comorbidity should be screened with caution, since a large proportion may harbor cancer that would be unlikely to affect their life expectancy.

The American Society of Clinical Oncology (ASCO) provisional clinical opinion recommends that in men with a life expectancy ≤ 10 years, PSA-based screening for prostate cancer should be discouraged; in men with a life expectancy longer than 10 years, clinicians should discuss the appropriateness of PSA-based screening with them (Basch et al., 2012).

## ADVISING PATIENTS ABOUT PROSTATE CANCER SCREENING

The advisory groups cited here are not helpful counselors for clinicians about screening for prostate cancer, since they vary in their expert opinions. However, they almost unanimously request shared decision-making before ordering the PSA test. Shared decision-making is a collaborative process between well-informed patients and their clinicians to make health-care decisions together by taking into account the best scientific evidence available, as well as the patients’ values and preferences (Informed Medical Decision, 2013). Shared decision-making involves the concept of autonomy and the ideas of self-determination, independence, and freedom. Patients have the right to accept or refuse PSA testing. 

Shared decision-making also obliges clinicians to take time to discuss the risks and benefits of PCA screening with their patients. Informed patients understand the nature and risk of prostate cancer, the risks, benefits, and alternatives to screening, make shared decisions to be screened or not at a level they desire, and make decisions consistent with their preferences and values (CDC, 2016). Clinicians’ understanding of the principle of autonomy will enable them to consider the patients as equal partners in the plan of care decision-making. Shared decision-making does not always take place between clinicians and patients.

Wheeler, Szymanski, Black, and Nelson (2011) provide some examples of impediments to shared decision-making for clinicians, such as a lack of time to educate patients about screening for prostate cancer, fear of malpractice litigation by failing to order the PSA test that could have helped detect cancer, and underestimating or overestimating the intellectual capacity of patients to participate in shared decision-making.

A family history of prostate cancer, African ancestry, and certain inherited genetic conditions are important risk factors of prostate cancer. A patient with a father or brother who developed prostate cancer is twice as likely to develop the disease. This risk is further increased if the cancer was diagnosed in family members at a younger age (up to 55 years of age) or if it affected three or more family members (Prostate Cancer Foundation, 2012).

The NCCN (2016) suggests that African American men compared with Caucasian American men have a higher incidence of prostate cancer, increased prostate cancer mortality, and earlier age of diagnosis. However, although these men may require a higher level of vigilance and different considerations when analyzing the results of screening tests, the panel cannot provide separate screening recommendations for these men until more data become available. The effects of earlier or more intensive screening on cancer outcomes and on screening-related harms in African American men remain unclear. The National Human Genome Research Institute (2012) reports that prostate cancer is more prevalent in African American men than in any other population and that scientists are focusing closely on the role of inherited factors.

The NCCN (2016) guidelines for genetic/familial high-risk assessment recommend that men with *BRCA1* or *BRCA2* mutations start prostate cancer screening at age 40. Men with a known history of *BRCA1/2* mutations should be referred to a cancer genetics professional, as they are at increased risk for prostate cancer associated with such mutations.

## CASE STUDIES

**Case Study 1**

Patient A is a 45-year-old Caucasian male. He has no history of cancer in his family and is asymptomatic.

Following the recommendations of the USPSTF, the AAFP, the NCI, the CDC, the ACS, and the AUA, clinicians may not recommend PSA-based screening for patient A. However, following the recommendations of the NCCN, the NCI, and the CDC, the risks and benefits of PSA-based screening for prostate cancer may be discussed with patient A for him to make an informed decision about PSA-based screening for prostate cancer.

**Case Study 2**

Patient B is a 45-year-old African American male. He has no history of cancer in his family and is asymptomatic.

Following the recommendations of the USPSTF, the AAFP, the NCI, the CDC, and the AUA, clinicians may not recommend PSA-based screening for patient B. However, following the recommendations of the NCCN, the AAFP, the NCI, the CDC, the ACS, and ASCO, the risks and benefits of PSA-based screening for prostate cancer should be discussed with patient B for him to make an informed decision about PSA-based screening for prostate cancer. 

**Case Study 3**

Patient C is a 75-year-old Caucasian male with a medical history of controlled asthma. He has no history of cancer in his family and is asymptomatic.

Following the recommendations of the USPSTF, the AAFP, and the AUA, clinicians may not recommend PSA-based screening for patient C. However, following the recommendations and guidelines of the NCCN, the NCI, the ACS, and ASCO, clinicians may discuss PSA-based screening with patient C.

**Case Study 4**

Patient D is a 60-year-old African American male. He is asymptomatic. His 62-year-old brother was successfully treated with radiation for prostate cancer. 

Following the recommendations of the AAFP, the NCI, the CDC, the ACS, the AUA, the NCCN, and ASCO, clinicians would find it less difficult to discuss PSA testing with patient D since he is an African American male with a first-degree relative diagnosed with prostate cancer before 65 years of age. However, following the recommendations of the USPSTF, clinicians may not recommend PSA-based screening for patient D. 

## CONCLUSION

Advising patients about screening for prostate cancer remains a challenge for clinicians, since expert guidelines and recommendations vary from various advisory groups. However, some clinicians will continue offering PSA testing, and some patients will continue requesting PSA testing. Nevertheless, clinicians should keep in mind that they should adopt the shared decision-making approach by discussing the potential risks and benefits of PSA-based testing with their patients before offering the test.
